# Integrating mutation and gene expression cross-sectional data to infer cancer progression

**DOI:** 10.1186/s12918-016-0255-6

**Published:** 2016-01-25

**Authors:** Julia L. Fleck, Ana B. Pavel, Christos G. Cassandras

**Affiliations:** Division of Systems Engineering, Boston University, 15 Saint Mary’s Street, Brookline, MA 02446 USA; Graduate Program in Bioinformatics, Boston University, 24 Cummington Mall, Boston, MA 02215 USA; Section of Computational Biomedicine, Boston University School of Medicine, 72 East Concord Street, Boston, MA 02118 USA

**Keywords:** Cancer progression, Somatic mutation, Gene expression, Mixed integer linear program, Breast cancer

## Abstract

**Background:**

A major problem in identifying the best therapeutic targets for cancer is the molecular heterogeneity of the disease. Cancer is often caused by an accumulation of mutations which produce irreversible damage to the cell’s control mechanisms of survival and proliferation. Different mutations may affect these cellular anachronisms through a combination of molecular interactions which may be dynamically changing during cancer progression. It has been previously shown that cancer accumulates mutations over time. In this paper we address the problem of cancer heterogeneity by modeling cancer progression using somatic mutation and gene expression cross-sectional data.

**Results:**

We propose a novel formulation of integrating somatic mutation and gene expression data to infer the temporal sequence of events from cross-sectional data. Using a mixed integer linear program we model the interaction between groups of different mutated genes and the resulting modifications at the gene expression level. Our approach identifies a partition of mutation events which gradually produce gene expression changes to a partition of genes over time. The proposed formulation is tested using both simulated data and real breast cancer data with matched somatic mutations and gene expression measurements from The Cancer Genome Atlas. First, we classify the genes as oncogenes or tumor suppressors based on the frequency of driver mutations. As expected, the most frequently mutated genes in breast cancer are PIK3CA and TP53 genes. Then, we select those genes with most frequent driver mutations and a set of genes known to play roles in cancer development. Furthermore, we apply the proposed mixed integer linear program to identify the temporal order in which genes mutate and, simultaneously, the changes they produce at the gene expression level during cancer progression. In addition, we are able to identify known causal relationships between mutations and gene expression changes in PI3K/AKT and TP53 pathways.

**Conclusions:**

This paper proposes a new model to infer the temporal sequence in which mutations occur and lead to changes at the gene expression level during cancer progression. The approach is general and can be applied to any data sets with available somatic mutations and gene expression measurements.

**Electronic supplementary material:**

The online version of this article (doi:10.1186/s12918-016-0255-6) contains supplementary material, which is available to authorized users.

## Background

Existing methods for identifying cancer specific markers and therapeutic targets typically analyze genetic, transcriptomics, proteomics or epigenetic data in search of common patterns. One of the biggest challenges in biomarker discovery is the heterogeneity of cancer data. Specific patterns discovered in one study often fail to validate in other studies or different data sets. The heterogeneity observed in clinical data may be due to (*i*) limitations associated with the platforms used for generating molecular data (e.g., sequencing, microarray, etc.), since batch effects are commonly observed between different platforms or protocols; (*ii*) limitations associated with existing cancer data sets, which are currently predominantely cross-sectional. This means that one time point measurements are generated for all patients, although such measurements may not necessarily be the most informative ones. In fact, certain genes dynamically change expression levels during the cell cycle, so that one time point measurements of gene expression may not capture the steady state expression level; (*iii*) imperfections of clinical diagnosis and the ensuing difficulty in assessing the stage of each patient’s tumor. As a result, clinical samples may not always be properly annotated; (*iv*) the reduced sample size with respect to the large number of measured features (genes or other molecules), which decreases the statistical power of the majority of commonly used approaches for biomarker discovery and patient stratification; (*v*) the heterogeneity of cancer mechanisms, which may also dynamically change during cancer progression.

In light of this, much of the research currently undertaken using cross-sectional data aims at determining whether the order in which genetic alterations occur within tumors follows common progression paths. Although not all patients with the same type of cancer harbor the exact same set of genetic abnormalities, there seems to be at least a subset of such changes that are consistent across a set of patients. This suggests that several combinations of mutations and gene expression patterns may lead to similar changes in cancer initiation and progression mechanisms such as apoptosis, differentiation, migration, and proliferation. In other words, different molecular patterns may contribute to cancer growth and proliferation in a similar way, i.e., through the deregulation of similar cellular mechanisms.

Much effort has recently been undertaken to collect, organize and make publicly available multiple data types obtained from genetic analysis of tumor samples. A case in point is the research developed by the The Cancer Genome Atlas (TCGA) Network, whereby different types of cancer have been profiled, such as breast invasive carcinoma [1], lung adenocarcinoma [2], lung squamous carcinoma [3], colon cancer [4], among others.

Existing data sets of cross-sectional data have been extensively used to derive temporal models capable of inferring sequences of mutational events and/or sequences of affected pathways responsible for driving cancer progression. In [5], Bayesian networks constructed using mutation data were used to infer the temporal order of genetic mutations. The concept of probability raising was applied to copy number variation (CNV) data to infer causal models of cancer progression in [6]. The model derived in [7] provided a quantitative understanding of the dynamics of tumorigenesis with respect to mutation, selection, genetic instability, and tissue architecture. The authors in [8] developed a model of somatic evolution of colorectal cancer based on published data and used it to investigate the effect of different parameters on tumor evolution on a global scale, while those in [9] suggested that stochastic dynamics alone might be responsible for either remission or rapid growth of tumors in the hematopoietic system.

The importance of connecting different types of genomic alterations (as opposed to a single type of genomic data) was recognized in [10] and [11], where probabilistic inference was used to predict the degree to which the activity of a pathway was altered in a given patient. For this purpose, each gene was modeled as a set of interconnected variables associated with expression, CNV and protein levels, and *a priori* information of molecular pathways was used to define the gene groups of interest and model the gene interactions.

Additional studies [12–14] have established a correlation between certain mutations and survival rates, thus revealing the genetic heterogeneity of cancer and the existence of multiple subtypes. Another example is the prognostic role of BRCA1 mutation in patients with triple-negative breast cancer [15].

Thus far, most attempts at reconstructing tumor progression at the pathway level have considered only known, *a priori* defined, pathways. The problem of simultaneously inferring the order of genetic mutation occurrence from somatic mutation cross-sectional data was formulated as an Integer Linear Program (ILP) in [16]. Unlike existing work, our approach neither correlates a single type of cross-sectional data (e.g., mutation, gene expression, etc.) with cancer prognosis, nor analyzes these different types of data separately to combine the resulting analyses *post-factum*. Instead we combine the information from somatic mutations and gene expression data using mathematical techniques and develop a model capable of inferring the chronological sequence of alterations at the genome as well as at the pathway level. Furthermore, our inference model is not restricted to *a priori* defined cellular pathways, but is also able to identify such pathways and the sequence in which these become altered during tumor evolution.

Our work builds upon the formulation proposed in [16] by first identifying the active cancer driver genes (oncogenes and tumor suppressors) in the data set using the approach from [17]. We address the issue of cancer heterogeneity by using both somatic mutation and gene expression data from cross-sectional measurements and thus propose a new Mixed Integer Linear Program (MILP) formulation to model the molecular progression of cancer. Our formulation is based on the assumptions that mutations accumulate over time [16, 18], and that different mutations may cause similar changes in the cell’s state, resulting in under/over expression of different groups of genes. Moreover, we consider that the progression of the disease is reflected in both the accumulation of mutations and changes in gene expression levels. Finally, we also assume that changes in gene expression may lead to the appearance of new mutations. We validate the MILP algorithm using simulated data and subsequently apply it to real cross-sectional breast cancer data. As a result, we are able to indentify *phases of cancer progression* that corroborate known interactions between genes in important breast cancer pathways. Of note, we analyze breast cancer data as a case study. However, our approach is general and easily applicable to other types of cancer.

This paper is organized as follows. In section ‘Methods’ we outline the MILP formulation, describe how it can be used to stratify molecular events such as mutation and gene expression changes, and discuss the data sets used in our analysis. Results obtained using both simulated data and real breast cancer data are presented in section ‘Results and discussion’. We conclude and discuss future work in section ‘Conclusions’.

## Methods

### Modeling cancer progression using a mixed integer linear program

The problem of partitioning mutational events into a temporal sequence of events was formulated in [16] as an Integer Linear Program (ILP), in which an optimal partition must satisfy two biologically meaningful requirements. The first, termed *exclusivity of mutations*, derives from the common assumption that at most one driver mutation takes place during each step (or *phase*) of cancer progression. The second one establishes *progression across phases* by enforcing that, for any given patient, a mutation in some gene must take place in a given progression *phase* in order for another gene to become mutated in a subsequent *phase*. The existence of passenger mutations, false positives and false negatives in mutation detection, among other factors, may lead to the violation of these requirements. Therefore, in order to enforce both exclusivity and progression, changes may need to be made to the original mutation data set.

In this paper paper we extend the model in [16] by adding the interaction between somatic mutations and gene expression. We propose a new Mixed Integer Linear Program (MILP) formulation which identifies the order in which mutations appear and produce changes of gene expression. In our approach, a *phase of cancer progression* is defined by both a group of *mutation genes* and a group of *expression genes*. We hypothesize that during a cancer progression *phase*, certain mutations lead to gene expression changes in multiple genes. Moreover, gene expression changes may cause mutations and expression changes in the downstream cancer progression *phases*. Our formulation is based on the following assumptions: (*A*1)*Exclusivity of driver mutations within each cancer progression phase.* This implies that each sample can have only one mutated gene and each gene can only be assigned to one *phase*; (*A*2)*Progression of mutations across subsequent phases*, so that each sample must have one gene mutated in the previous *phase* in order to have a mutation in a subsequent *phase*; (*A*3)*Causality relationship between mutated genes and genes with abnormal expression.* Mutations in driver genes lead to changes in expression of certain genes. Hence, if a sample has no mutated genes in a given *phase*, all genes in the expression subset of that *phase* must have normal expression; (*A*4)*The strength of the connection between expression and mutation genes determines the assignment of the abnormal expression genes to the corresponding phases.* This means that each expression gene is assigned to a certain *phase* based on the strength of this gene’s connection to the *mutation genes* that belong to that *phase*.

Our input data consists of an *m*×*n* binary mutation matrix *M*, as well as an *m*×*r* expression matrix *E*, where *m* is the number of samples (patients) in our database, *n* is the number of mutation genes considered in our study, and *r* is the number of expression genes considered in our study. The values of the entries *M*_*ij*_ of the mutation matrix are

$$M_{ij}=\left\{ \begin{array}{ll} 1 & \text{if mutation gene}\; j \; \text{is mutated in sample}\;i \\ 0 & \text{otherwise} \end{array} \right. $$

while the entries *E*_*ih*_ of the expression matrix correspond to the measured expression level of expression gene *h* for sample *i*. We define the *connectivity* between mutation gene *j* and expression gene *h* to be the product between the mutation status of gene *j* and the expression level of gene *h*, compounded across all samples. Hence, we construct an *r*×*n* real-valued connectivity matrix *C*≡*E*^*T*^·*M*. The value of entry *C*_*hj*_ of the connectivity matrix can be interpreted as follows: values closer to zero indicate that most samples exhibit small absolute values of expression levels for gene *h* and/or have no mutation in gene *j*; conversely, the further away the value of *C*_*hj*_ is from zero, the stronger is the connectivity between expression gene *h* and mutation gene *j* across the data set.

It is thus clear that a stronger connectivity is obtained when $\left \vert E_{ih}\right \vert \gg \frac {1}{m}\underset {i=1}{\overset {m}{\sum }}E_{ih}$ and *M*_*ij*_=1 for *i*=1,…,*m* and ∀ pair $\left ({h,j}\right) $ of expression and mutation genes. Note that, in what concerns the expression levels, this condition implies that the most under/over expressed genes yield higher connectivity scores. This in turn points to the relevance of preprocessing matrix *E* so as to identify abnormally low/high *E*_*ih*_ values. Such task can be easily accomplished through outlier detection techniques. While outliers are typically associated with erroneous data, in this case values of *E*_*ih*_ that deviate markedly from the mean expression level are particularly relevant. Indeed, our ultimate goal is to infer sequences of abnormal cellular behavior that lead to cancer progression, which means that we are interested in analyzing genes that exhibit mutations and/or abnormal expression levels. As a result, we performed a percentile analysis for each expression gene *h*, *h*=1,…,*r* and considered entry *E*_*ih*_ to be over (under, respectively) expressed if it belonged to the 99^*th*^ (1^*st*^, respectively) percentile of gene *h*. As a final preprocessing step, we modified the values of all entries *E*_*ih*_ so as to generate a binary expression matrix, where the value of 1 indicated that sample *i* belonged to either the 1^*st*^ or 99^*th*^ percentile of gene *h*, and the value of zero indicated otherwise.

In this context, the problem of inferring a model of cancer progression can be cast as that of finding a partition of the *n* columns of matrix *M* into *K* mutation *phases* and a partition of the *r* columns of matrix *E* into *K* expression *phases*. We address the role of the number of *phasesK* in a subsequent discussion, but note that the value of *K* is externally selected and varies depending on the desired number of *phases*. Intuitively, *K* reflects the level of abstraction of the model: a large (small, respectively) value of *K* corresponds to a microscopic (macroscopic, respectively) model. However, for the problem we consider here, it is not reasonable to assume that a microscopic model is necessarily superior to a macroscopic one, hence the need to vary the value of *K* and scrutinize the corresponding results. In this context, our problem can be formulated as the following MILP:

(1) $$  {{\begin{aligned} \min {\left[ \frac{1-W}{m\cdot n}\sum\limits_{i=1}^{m} \sum\limits_{k=1}^{K} \left(\sum\limits_{j=1}^{n} M_{i,j} p_{j,k}^{M} - a_{i,k}^{M} + 2f_{i,k}^{M}\right)\! -\! \frac{W}{K\cdot r} \sum\limits_{k=1}^{K} \sum\limits_{h=1}^{r} p_{h,k}^{E}\right] } \end{aligned}}}  $$

$$ {{\begin{array}{llll} s.t. & \sum_{k=1}^{K}{p_{j,k}^{M}}=1 & \forall\ \text{mutation gene}\ j & \!(C1)\\ & \sum_{k=1}^{K}{p_{h,k}^{E}}\geq 0 & \forall\ \text{expression gene}\ h &\! (C2) \\ & \sum_{j=1}^{n}{p_{j,k}^{M}}\geq 1 & \forall\ \text{phase}\ k & \!(C3) \\ & \sum_{h=1}^{r}{p_{h,k}^{E}}\geq 0 & \forall\ \text{phase}\ k & \!(C4) \\ & a_{i,k}^{M}\geq a_{i,k+1}^{M} & \forall\ \text{sample}\ i,\forall\ \text{phase}\ k & \!(C5) \\ & a_{i,k}^{M}\leq f_{i,k}^{M}+\sum_{j=1}^{n}{M_{i,j}\cdot p_{j,k}^{M}} & \forall\ \text{sample}\ i,\forall\ \text{phase}\ k & \!(C6) \\ & p_{h,k}^{E}=\sum_{j=1}^{n}{C_{h,j}\cdot p_{j,k}^{M}} & \forall \ \text{expression gene}\ h,\forall \ \text{phase}\ k & \!(C7) \end{array}}} $$

where the optimization is performed over variables $p_{{j,k}}^{M}$, $f_{{i,k}}^{M}$, and $a_{{i,k}}^{M}$, which all take values in {0,1} such that $p_{{j,k}}^{M}=1$ if mutation gene *j* is assigned to *phaseP*_*k*_; $f_{{i,k}}^{M}=1$ if we need to flip one entry of columns in *phasek* in order for *phasek* to be mutated in sample *i*; $a_{{i,k}}^{M}=1$ if sample *i* is considered mutated in *phasek* after any required flips. We also optimize over variable $p_{{h,k}}^{E}\in \left [ 0,1\right ] $, which is the probability of expression gene *h* being assigned to *phasek*.

The objective function in (1) contains two terms, the first of which was proposed in [16]. As mentioned previously, several factors may lead to the violation of constraints (*C*1)−(*C*6), which means that it may be necessary to alter the mutation matrix *M* by flipping some of its entries from 0 to 1 (non-mutated to mutated) or 1 to 0 (mutated to non-mutated). In this context, the first term of the objective function corresponds to the number of entries of matrix *M* that need to be flipped, which should be minimized. For the sake of completeness, we provide here a brief interpretation of this term, and the reader is referred to [16] for further details. For a given sample *i*, *i*=1,…,*m* and *phasek*, *k*=1,…,*K*, once the values of variables $p_{{j,k}}^{M}$, $f_{{i,k}}^{M}$, and $a_{{i,k}}^{M}$ have been fixed, the contribution of each sample *i* to the objective function corresponds to the number of entries in *phasek* that are flipped in sample *i*. Since two types of flips are possible (i.e., $f_{{i,k}}^{M}=1$ if either a 0 to 1 flip or a 1 to 0 flip is performed), this number is given by $\sum _{j=1}^{n} M_{{i,j}} p_{{j,k}}^{M}- a_{{i,k}}^{M} + 2f_{{i,k}}^{M}$. The second term of the objective function, which we seek to maximize, compounds the probability of expression gene *h* being assigned to *phasek*, for *h*=1,…,*r* and *k*=1,…,*K*.

Note that because we define our objective function as a combination of objectives, it is necessary to ensure that each objective (i.e., each term of the objective function) is properly normalized. For each term, a normalization factor was defined as an upper bound on the corresponding objective component. It is simple to verify that, for the first term, the upper bound on the number of flips that could potentially need to be made corresponds to the total number of elements in matrix *M*, which is given by the product *m*·*n*. In the case of the second term, since $p_{{h,k}}^{E}$ is compounded across all expression genes and all *phases*, the natural normalization factor is simply given by the product *r*·*K*. Recall that we define variable $p_{{h,k}}^{E}$ as the probability of expression gene *h* being assigned to *phasek*, so that it is also necessary to scale its value to the range $\left [ 0,1\right ] $. From $\left (C7\right) $, it can be seen that variable $p_{{h,k}}^{E}$ is a combination of matrix *C* and binary variable $p_{{j,k}}^{M}$. The latter is naturally normalized, and the entries *C*_*hj*_ of the former can be scaled as follows, for *h*=1,…,*r*:

$$C_{hj}=\frac{C_{hj}}{\sum_{j=1}^{n}{C_{h,j}}} $$

Additionally, it can be seen that (1) includes a weight *W* associated with the second objective component. In order to ensure that the objective function is a convex combination of objectives, we associate a weight 1−*W* with the first term. It is also important to mention that these weights are not necessary for the purposes of normalization, but that their values can be chosen so as to ultimately reflect a desired trade-off (e.g., more importance can be assigned to the mutation data by setting *W*<0.5, and vice-versa for the gene expression data).

Finally, we briefly discuss the interpretation of constraints (*C*1)−(*C*7). The first constraint ensures that each mutation gene is assigned to exactly one cancer progression *phase*, while constraint (*C*2) enforces the assignment of each expression gene to at least one cancer progression *phase*. Moreover, any *phase* must consist of at least one mutated gene, but may have no expression genes assigned to it (constraints (*C*3) and (*C*4), respectively). Progression of mutational events is ensured in the fifth constraint, whereby sample *i* must have a certain mutated gene assigned to *phasek*, in order for this same sample to have another mutated gene assigned to *phasek*+1. Constraint $\left (C6\right) $ simply enforces the fact that, if sample *i* has a given mutated gene *j* assigned to *phasek*, then this gene is either already mutated in the original mutation matrix *M*, or its mutation status is a result of a 0 $\rightarrow $ 1 mutation flip. The last constraint ensures that the assignment of each expression gene *h* to any *phasek* is determined in terms of the corresponding probability $p_{{h,k}}^{E}$.

**Fig. 1:**
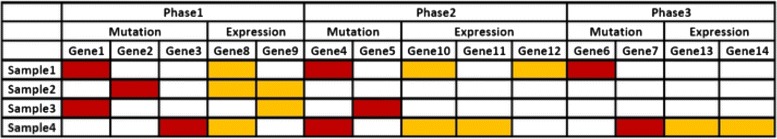
Example of a feasible solution of the MILP formulation proposed in this paper. Red boxes represent genes with mutation. Orange boxes mark genes with altered gene expression levels. White boxes correspond to those entries with no mutations or no expression changes

We end by noting that, while exclusivity of driver mutations within each cancer progression *phase* is enforced in our formulation, exclusivity of changes in expression levels is not. As a result, for any given sample *i*, more than one expression gene *h* may be assigned to *phasek*. Nevertheless, our formulation enforces a temporal association between mutational events and changes in gene expression. In other words, for any given sample *i*, no expression genes may be assigned to *phasek*, unless a given mutation gene *j* has been assigned to this*phase*. For illustrative purposes, an example of a feasible solution for the proposed MILP is presented in Fig. 1.

### Data

We test the approach by using both simulated data and real cancer data. We apply our model on the breast invasive carcinoma (BRCA) data set generated by the The Cancer Genome Atlas (TCGA) Research Network. Publicly available somatic mutation data (level 2) and gene expression data (level 3) was downloaded from TCGA [19]. The somatic mutations data was profiled for 993 subjects by Whole-Exome Sequencing on Illumina GA DNA Sequencing platform. Gene expression generated on UNC Agilent G4502A was profiled for 547 subjects [1]. For our analysis, we consider 529 subjects with both types of data measurements (somatic mutations and gene expression).

Our approach is general and we can infer the sequence of events for any gene sets. However, in this paper we consider genes that are more relevant to breast cancer. To select the relevant cancer driver genes based on their mutation frequency we use the *20/20 rule* proposed in [17] which classifies genes into oncogenes or tumor suppressors.

## Results and discussion

In what follows we present the results obtained by applying our methodology to both simulated data and real patient data from breast cancer studies. In all cases the MILP was solved using CPLEX v12.6 with default parameters.

### Simulated data

We performed an experiment using simulated data to illustrate the desired behavior of our model. For such, we used an *m*×*n* binary simulated mutation matrix *M*^*S*^ and an *r*×*n* real-valued simulated connectivity matrix *C*^*S*^, where *m* is the number of samples in our data set, *n* is the number of mutation genes, and *r* is the number of expression genes. For simplicity, and without loss of generality, we take *M*^*S*^ to be the mutation matrix obtained from the TCGA breast cancer data set, where *m*=529 samples and *n*=72 mutation genes (as described in section ‘TCGA breast cancer data’). The purpose of our simulation experiments is to show that our MILP model is capable of correctly extracting the information contained in mutation and gene expression data regarding which expression genes are more strongly connected to which mutation genes. Hence, we arbitrarily assigned values to the entries $c_{hj}^{S}$ of matrix *C*^*S*^ such that $\underset {j=1}{\overset {n}{\sum }}c_{hj}^{S}=1$, for *h*=1,…,*r*. This condition enforces an extreme scenario in which each expression gene is only connected to a single mutation gene. Thus, the expected outcome of applying our MILP model to such data is that each expression gene should be uniquely assigned to the same *phase* as the mutation gene to which it is connected. For simplicity, and also without loss of generality, we took *r*=319 expression genes, so as to make the dimensions of the simulated data compatible with those of the real data we analyze.

Three simulation runs were performed by varying the number of *phasesK* such that $K\in \left \{ 2,3,4\right \} $, and the corresponding results are shown in Tables 1, 2, 3. In all cases, our results showed that expression genes were partitioned in complete agreement with the connectivity data contained in *C*^*S*^. In other words, each expression gene *h*, *h*=1,…,*r*, was assigned to *phasek*, *k*=1,…,*K*, iff $c_{hj}^{S}=1$ and mutation gene *j*, *j*=1,…,*n*, was also assigned to *phasek*. For example, by analyzing Table 3 and the data in matrix *C*^*S*^, it is possible to verify that the 101 expression genes in *phasek*=1 are precisely the same ones that are connected to the mutation genes assigned to *phase* 1 (a similar analysis also holds for *phasesk*=2,3,4).

**Table 1:** Number of mutation and expression genes assigned to each *phase* of cancer progression using simulated data for K=2

Phase (*k*)	Number of mutation genes	Number of expression genes
1	39	175
2	33	144
**T** **O** **T** **A** **L**	**72**	**319**

The total number of genes is highlighted in bold

**Table 2:** Number of mutation and expression genes assigned to each *phase* of cancer progression using simulated data for K =3

Phase (*k*)	Number of mutation genes	Number of expression genes
1	26	117
2	25	111
3	21	91
**T** **O** **T** **A** **L**	**72**	**319**

The total number of genes is highlighted in bold

**Table 3:** Number of mutation and expression genes assigned to each *phase* of cancer progression using simulated data for K =4

Phase (*k*)	Number of mutation genes	Number of expression genes
1	22	101
2	22	99
3	19	82
4	9	37
**T** **O** **T** **A** **L**	**72**	**319**

The total number of genes is highlighted in bold

### TCGA breast cancer data

#### Selecting relevant cancer genes

In order to determine the sequence of somatic mutation and gene expression changes, we first narrow down the mutation and expression gene sets to interesting breast cancer genes. In [16] the authors consider all present mutations. However, since many somatic mutations are passenger and do not impact cancer progression, we first select those genes that are more likely to be drivers. Moreover, some genes are known to be involved in cancer associated cell processes, such as proliferation, migration and apoptosis. We are interested to see how the expression of these genes is affected by the driver mutation and which is the temporal order of changes that occur during cancer progression.

To select the relevant cancer driver genes based on their mutation frequency, we first classify genes into oncogenes or tumor suppressors by using the *20/20 rule* [17]. This method takes into account particular types of mutations and their frequencies. First, for a given gene, the total number of variants is computed across the data set. Then, each gene is assigned an oncogene (ONG) score and a tumor suppressor gene (TSG) score which are computed based on the frequency of gain-of-function or loss-of-function mutations, respectively. Gain-of-function mutations are defined as missense or in-frame indels that are recurrently mutated at the same aminoacid position, while loss-of-function mutations are nonstop, nonsense and frameshift indels [17]. For each gene, the ONG score is the frequency of gain-of-function mutations out of the total number of variants, while the TSG score is the frequency of all loss-of-function mutations out of the total number of variants. If the ONG score is greater than 20 %, then the gene is classified as an oncogene. Similarly, if the TSG score is higher than 20 %, then the gene is classified as a tumor suppressor.

We consider a gene to be a potential cancer driver if it presents mutations across the data set (a minimum number of 20) and has an ONG or a TSG score greater than 20 %. Based on this criterion, 72 genes are selected. The list of 72 genes include genes that were previously found to be highly mutated in breast cancer, such as: PIK3CA, PTEN, TP53, GATA3, CDH1, RB1, MLL3, MAP3K1, TBX3, RUNX1, CBFB, NF1 [1].

To infer the *cancer progression phases* of gene expression changes we consider the *Pathways in Cancer* set from the Kyoto Encyclopedia of Genes and Genomes database (KEGG) [20, 21]. After overlapping this set with our data, we obtain 319 genes which are known to play a role in cancer initiation and progression.

Therefore, we consider two sets of genes: genes that present driver mutations and genes implicated in cancer development.

Finally, we wish to infer the stages in which mutations occur and the relations between mutational events and gene expression changes. For this, we apply the proposed MILP model as detailed next.

#### Identifying a temporal sequence of mutation events and gene expression changes for breast cancer

**Fig. 2:**
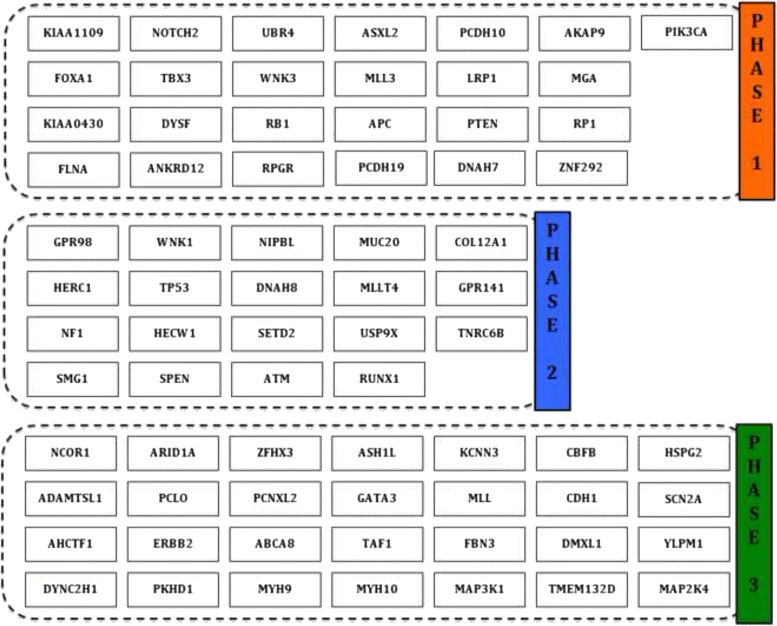
Optimal solution of the MILP algorithm for cancer mutation and expression genes (K =3). Shown here is the assignment of mutation genes to each *phase of cancer progression*

**Fig. 3:**
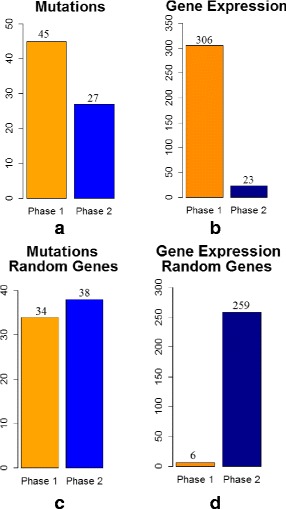
Number of genes assigned to each *phase of cancer progression* for K = 2 (**a**) cancer mutation genes; (**b**) cancer expression genes; (**c**) random mutation genes; (**d**) random expression genes: 20 % of genes were not assigned to any *phase*

**Fig. 4:**
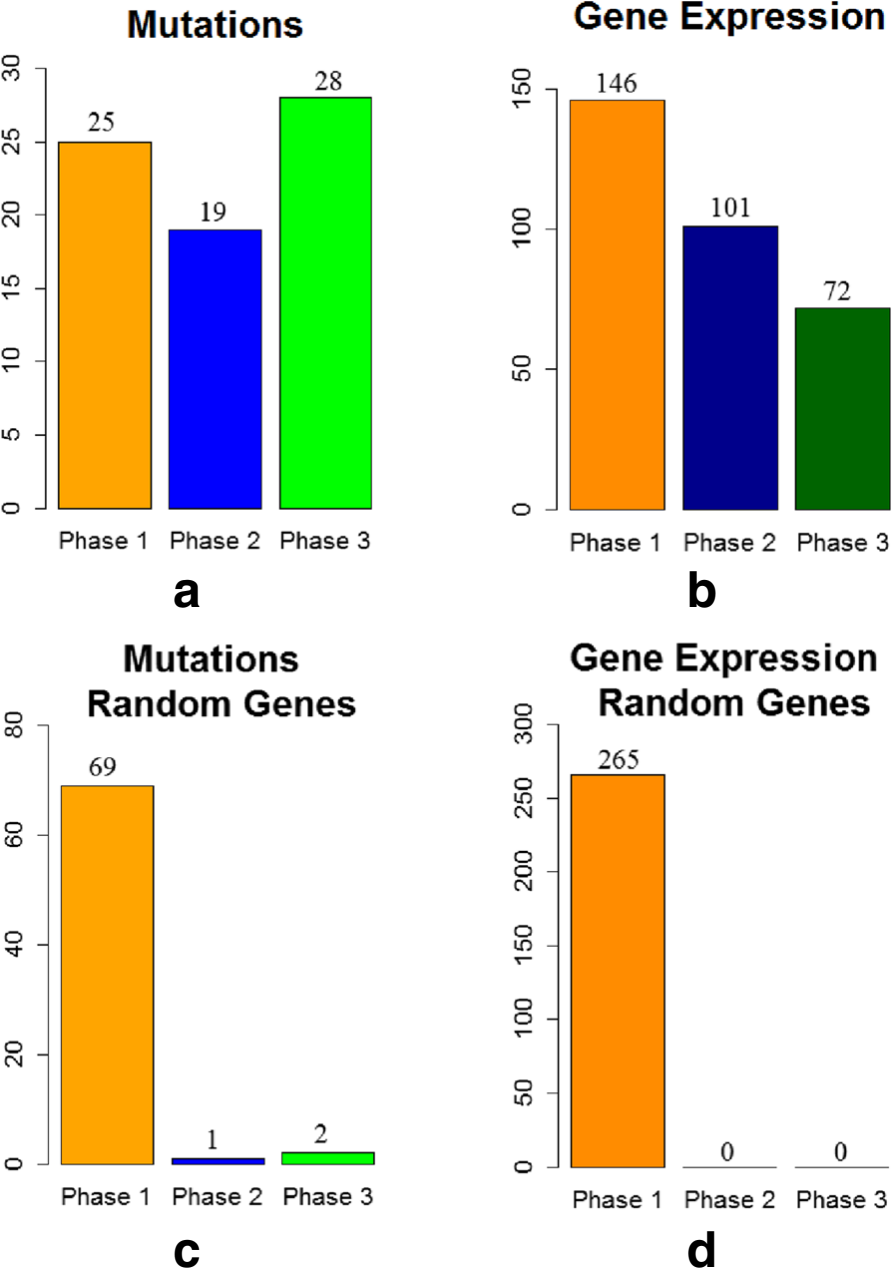
Number of genes assigned to each *phase of cancer progression* for K = 3 (**a**) cancer mutation genes; (**b**) cancer expression genes; (**c**) random mutation genes; (**d**) random expression genes: 20 % of genes were not assigned to any *phase*

**Fig. 5:**
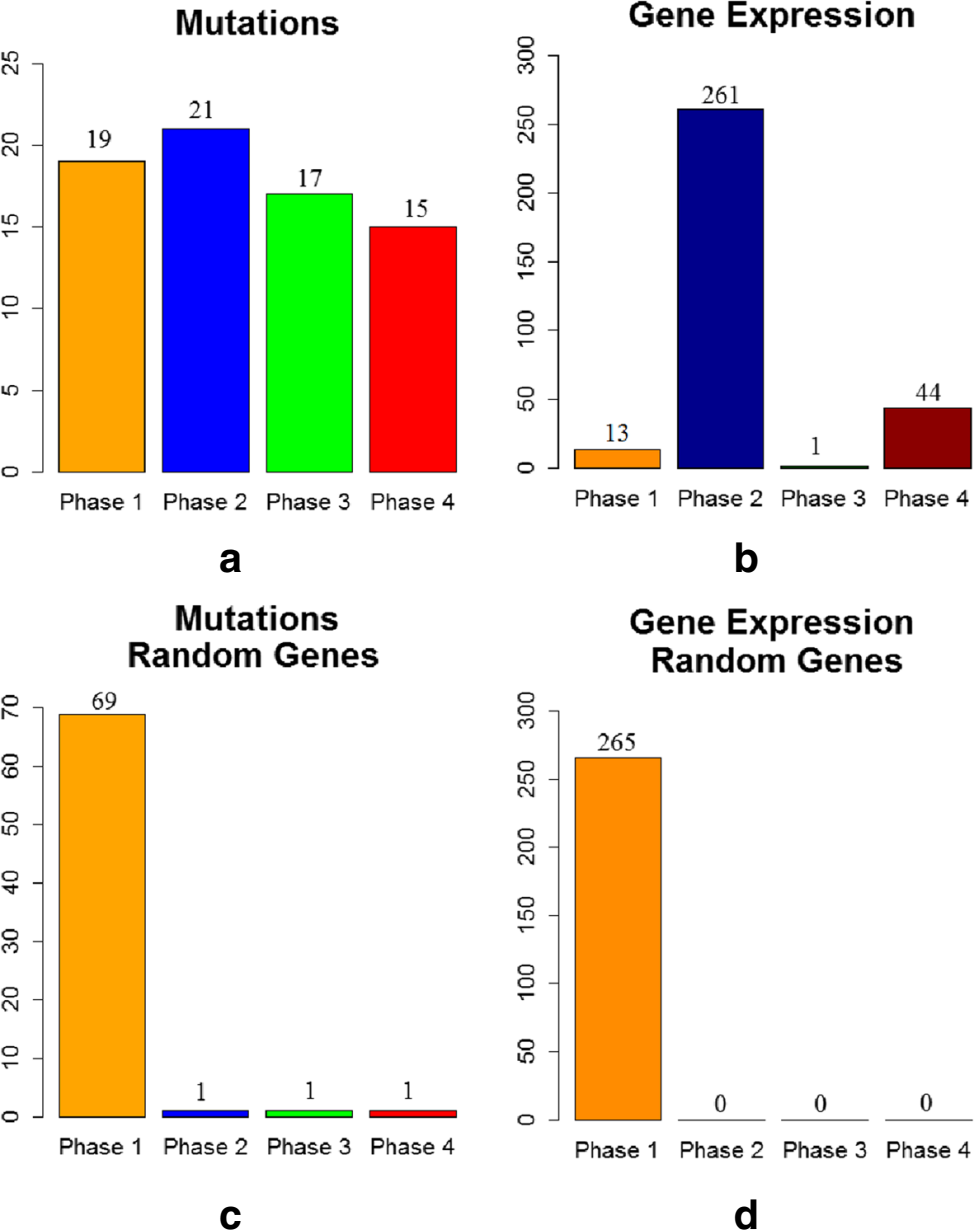
Number of genes assigned to each *phase of cancer progression* for K = 4 (**a**) cancer mutation genes; (**b**) cancer expression genes; (**c**) random mutation genes; (**d**) random expression genes: 20 % of genes were not assigned to any *phase*

**Fig. 6:**
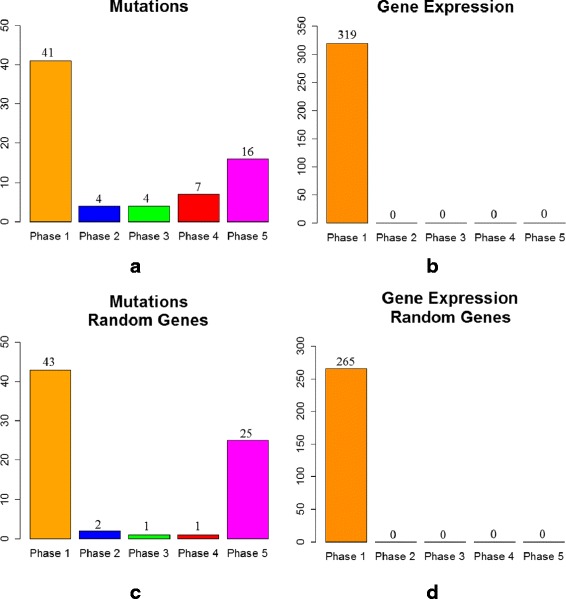
Number of genes assigned to each *phase of cancer progression* for K = 5 (**a**) cancer mutation genes; (**b**) cancer expression genes; (**c**) random mutation genes; (**d**) random expression genes: 20 % of genes were not assigned to any *phase*

We applied the proposed MILP formulation with a different number of predefined *phasesK*=2,3,4,5 and selected the most biologically meaningful one. For a number of 3 *phases*, the algorithm was able to stratify the mutation and expression genes in different proportions within each *phase*. For *K*=2,4,5, most of the expression genes were placed in one *phase* and the results do not reflect a gradual progression. Figures 3(a), (b), 4(a), (b), 5(a), (b), and 6(a), (b) illustrate the number of genes assigned to each *phase* for the expression and mutation groups. For a number of 3 *phases*, the mutation genes are more or less evenly distributed across the 3 *phases* (Fig. 4(a)). The mutation genes of each *phase* are shown in Fig. 2. In addition, one can notice that the number of gene expression modifications gradually decrease from *phase* 1 to *phase* 3 (Fig. 4(b)). As expected, more cancer genes present abnormal expression under the influence of earlier stage mutations, such as PIK3CA and PTEN, since these genes are important in cancer initiation.

The proposed 3-*phase* sequence of events in breast cancer, for the two sets of 72 mutation genes and 319 expression genes can be found in Additional file 1.

Furthermore, we validate our results by running similar experiments of different number of *phases*, on the same number of arbitrarily selected genes, both for mutation and expression sets (Figs. 3(c), (d), 4(c), (d), 5(c), (d), and 6(c), (d)). By randomly selecting these genes from the entire data set of 17814 genes, not all of them will particularly be associated with cancer events. Therefore, these experiments will serve as a negative control. As expected, in all of the experiments we ran on the randomly selected genes, the MILP assigns most of the genes to one *phase*, not being able to find a temporal sequence of events. In addition, 20 % of the expression genes are not assigned to any *phase*. Figures 4(c) and 4(d) illustrate the random results for *K*=3*phases* using random gene sets. One can notice the difference between the progression of cancer genes compared to random genes.

The idea of comparing the results generated on the genes of interest with those on a randomly selected set of genes could serve as a general approach for selecting *K*. The adequate value for *K* will most likely change when different cancer data sets or gene sets are used. Therefore, it is important to select the best configuration as the one that gives the most biological meaningful results and that also differs from the negative control. This approach for selecting *K* can generally be applied to any cancer data set, as long as it is also combined with biological insight about the analyzed set of genes.

For any given *phase* of mutation and expression genes, a patient has one and only one mutated gene during each *phase* which is associated to one or more genes with abnormal gene expression level. Therefore, our method is able to stratify the heterogeneity of mutations and gene expression changes into a temporal order of events. This crucial observation points to the contribution of this work, but it also brings to light the issue of uniqueness of solution of the proposed MILP formulation, which we briefly address here. Note that the existence of a single optimal solution to our problem indicates that it is possible to find a unique configuration of *phases* that optimally satisfies our formulation. On the other hand, the lack of a unique optimum means that several equivalent solutions could potentially be identified, and that different configurations of *phases* could yield similar results. More importantly, either scenario (unique or multiple optimal solutions) may bring new insights for understanding the mechanisms of cancer development. In what follows, we present the insights provided by the results reported in this work.

**Fig. 7:**
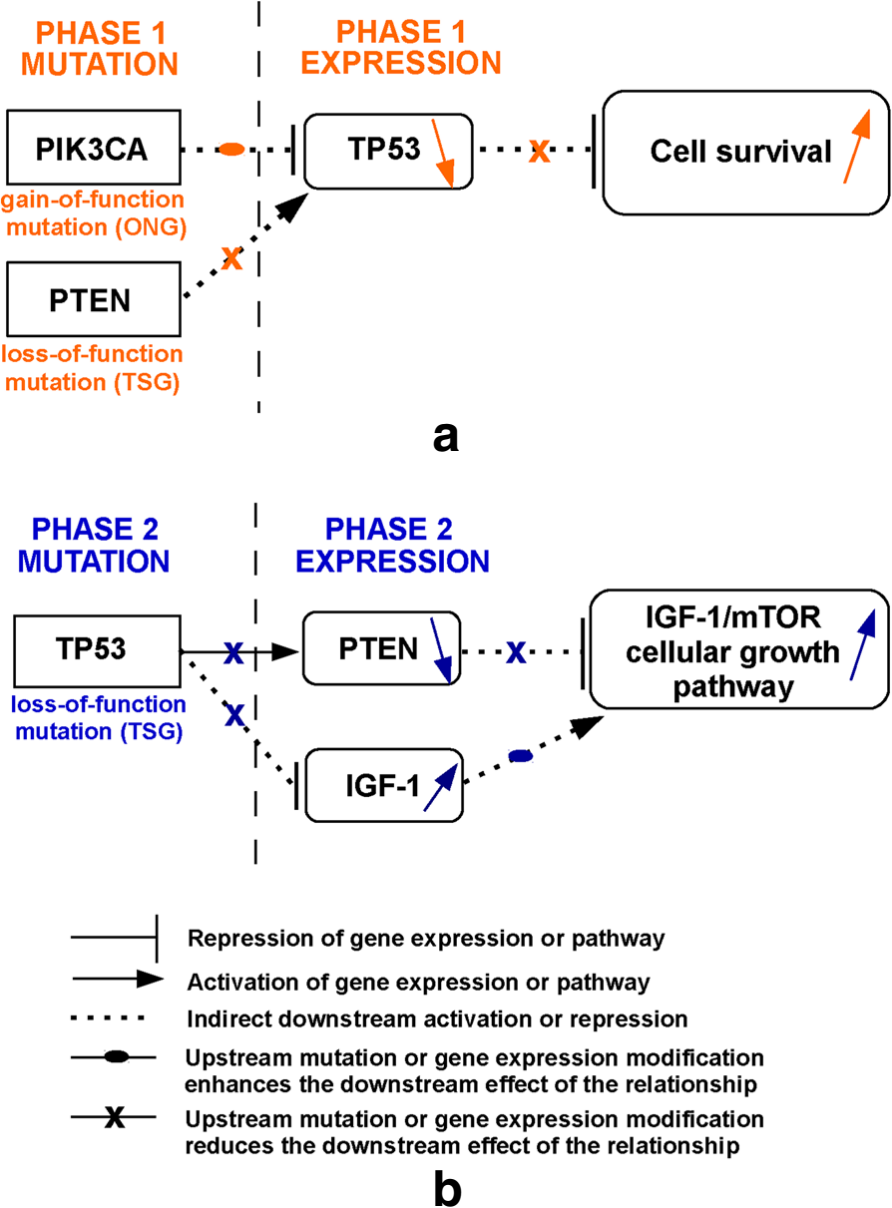
MILP model identifies causal relationships from PI3K/AKT and TP53 pathways (KEGG) (**a**) PI3K/AKT pathway is altered in *phase* 1 of breast cancer progression; (**b**) TP53 pathway is altered in *phase* 2 of breast cancer progression

We begin by evaluating the proposed partition so as to identify known causal gene relationships from cancer pathways, such as PI3K/AKT and TP53 from KEGG [21]. Figure 7(a) shows such interactions which occur in *phase* 1. PIK3CA is an oncogenic driver which is highly mutated in breast cancer (the ONG score computed based on the method in [17] is 90 % compared to a TSG score of 0.5 %). Also, PTEN gene presents significant loss-of-function mutations (the TSG score is 51 % compared to an ONG score of 5 %). We find PIK3CA and PTEN as being mutated in early stage during *phase* 1. Also, the events in *phase* 1 produce abnormal gene expression changes of TP53. TP53 is a well known tumor suppressor [22–24], situated downstream PIK3CA and PTEN in PI3K/AKT pathway. Mutations in PIK3CA or PTEN genes may decrease the gene expression level of TP53 tumor suppressor through AKT/MDM2 cascade. Consequently, low expression of TP53 may induce cell survival (Fig. 7(a)).

Next, we evaluate the number of patients that present a potentially active PI3K/AKT pathway. We find 20 % of patients to have mutations in PIK3CA, as well as decreased TP53 expression. In addition, about 2 % of patients present PTEN mutations, as well as decreased TP53 expression (Fig. 7(a)). In order to estimate if a gene is over- or under-expressed we compared the log normalized gene expression to 0 (negative values indicate under-expression in respect to the normal level, while positive values indicate over-expression in respect to the normal level).

Moreover, we identify changes in TP53 pathway during *phase* 2 of progression, as shown in Fig. 7(b). Mutations in TP53 gene generally produce the loss-of-function of its tumor suppressor activity [23]. Loss-of-function of TP53 gene may cause abnormal gene expression levels of the downstream genes, such as PTEN and IGF-1, which may activate the IGF1-mTOR cellular growth pathway and inhibit apoptosis. As expected PTEN and IGF-1 are assigned to *phase* 2 *expression genes* of the proposed 3-*phase* partition.

We find 29 % of patients to have a mutation in TP53 gene and increased IGF-1 expression. Moreover, 6 % of patients present a TP53 mutation, as well as both increased IGF-1 expression and decreased PTEN expression (Fig. 7(b)).

Interestingly, about 5 % of patients present mechanisms of both PI3K/AKT and TP53 pathways. They have a mutation in PIK3CA gene and decreased TP53 expression (Fig. 7(a)). In addition, they present mutations in TP53 gene and increased IGF-1 expression (Fig. 7(b)). Based on our approach, we are able to infer that mutations in PIK3CA (*phase* 1) precede the mutations in TP53 (*phase* 2).

## Conclusions

In this paper we propose a novel approach of integrating somatic mutations with gene expression data to infer the temporal sequence of mutation events and gene expression changes during cancer progression.

First, we validate the model using simulated data. Second, we apply the approach on breast cancer data from TCGA. We identify the temporal order of molecular changes of 72 most highly mutated driver genes in breast cancer data set and 319 cancer associated genes from KEGG *Pathways in Cancer* gene set. Moreover, we identify known gene relationships from PI3K/AKT and TP53 pathways.

Our approach is general and can be applied to other sets of genes of interest, as well as to other types of cancer. Larger sets of genes that potentially play a role in cancer progression can be analyzed. The temporal sequence of events also illustrates the causal relationships between potential mutation events which occur during a phase and the consequent expression changes during that phase. This information could be used for developing efficient drug combinations which target a specific group of genes that cause important expression changes. As future work we plan to further evaluate the method in other cancer data sets that provide somatic mutations and gene expression measurements of the same patients, such as TCGA colorectal cancer, glioblastoma, lung squamous cell carcinoma and ovarian cancer.

Our model identifies the groups of genes which change during cancer progression from cross-sectional data. Moreover, it offers new insights for understanding the heterogeneity of cancer mechanisms which are reflected by different combinations of mutations and gene expression changes. Our framework can be used to further address clinical questions and improve therapeutic strategies, such as the development of early detection biomarkers and efficient drug combinations.

## Availability of supporting data

The results published in this paper are in part based upon the publicly available data generated by the TCGA Research Network: http://cancergenome.nih.gov/. The Somatic Mutations and Expression-Genes data sets of Breast invasive carcinoma (BRCA) [1] (doi:10.1038/nature11412) have been downloaded from the TCGA Portal.

## Additional file

Additional file 1
**The partition of 72** ***mutation*** **and 319** ***expression genes*** **in 3 phases of cancer progression.** The values of the *expression genes* represent the likelihood score corresponding to each *phase*. (PDF 47.1 kb)

## Abbreviations

BRCA: breast invasive carcinoma
CNV: copy number variation
ILP: integer linear program
KEGG: kyoto encyclopedia of genes and genomes
MILP: mixed integer linear program
ONG: oncogene
TCGA: the cancer genome atlas
TSG: tumor suppressor gene
