# Supplementary material for: Integrating mutation and gene expression cross-sectional data to infer cancer progression
Source: BMC Syst Biol. 2016 Jan 25;10:12. doi: 10.1186/s12918-016-0255-6 (PMC4727329; doi:10.1186/s12918-016-0255-6)
Supplement: Additional file 1 — The partition of 72 mutation and 319 expression genes in 3 phases of cancer progression. The values of the expression genes represent the likelihood score corresponding to each phase. (PDF 47.1 kb) [file 12918_2016_255_MOESM1_ESM.pdf]

MUTATION GENES		
Phase 1	Phase 2	Phase 3
KIAA1109	GPR98	NCOR1
FOXA1	WNK1	ARID1A
NOTCH2	NIPBL	ZFHX3
TBX3	MUC20	ASH1L
UBR4	COL12A1	KCNN3
WNK3	RUNX1	MAP3K1
ASXL2	ATM	CBFB
MLL3	SMG1	TMEM132D
PCDH10	HERC1	ADAMTSL1
LRP1	TP53	PCLO
AKAP9	DNAH8	PCNXL2
MGA	MLLT4	GATA3
DNAH7	GPR141	MLL
PIK3CA	TNRC6B	CDH1
ANKRD12	USP9X	SCN2A
RPGR	SPEN	MAP2K4
KIAA0430	NF1	AHCTF1
DYSF	HECW1	ERBB2

RB1	SETD2	ABCA8
APC		TAF1
PTEN		FBN3
RP1		DMXL1
ZNF292		YLPM1
PCDH19		HSPG2
FLNA		DYNC2H1
		PKHD1
		MYH9
		MYH10

EXPRESSION GENES			
Genes	Phase 1	Phase 2	Phase 3
HRAS	0.2	0.35	0.45
PGF	0.3333	0.2857	0.3809
PTGS2	0.3701	0.3702	0.2591
STAT5A	0.3746	0.3434	0.2809
STAT5B	0.2332	0.3998	0.3665
SHH	0.452	0.3551	0.1937
CTNNB1	0.3914	0.3914	0.2175
CUL2	0.362	0.4258	0.213
PIK3CA	0.3751	0.4167	0.2085
RARA	0.25	0.5	0.25
FAS	0.4003	0.3431	0.2574
RARB	0.259	0.4442	0.296
CCNA1	0.5002	0.1667	0.3334
PIK3CG	0.4375	0.375	0.1875
WNT10A	0.2779	0.4446	0.2779
PLD1	0.4117	0.5293	0.0588
WNT10B	0.3876	0.2856	0.3264
BCR	0.2665	0.3666	0.3664
BRAF	0.3144	0.2572	0.4287
PIK3CB	0.3635	0.318	0.3179
RXRB	0.3125	0.125	0.5625
RXRA	0.28	0.44	0.28
PIK3CD	0.1666	0.5832	0.2499
SKP2	0.4168	0.3751	0.2084
RXRG	0.4545	0.3636	0.1818
CTNNA1	0.2665	0.4665	0.2664
CTNNA3	0.3683	0.2631	0.3683

CTNNA2	0.4349	0.2609	0.3045
VEGFB	0.4447	0.1111	0.4448
JUP	0.4617	0.2693	0.2695
MAPK1	0.2693	0.3078	0.4233
VEGFC	0.1539	0.4233	0.4234
PIAS4	0.238	0.4286	0.3332
LAMC3	0.3333	0.3333	0.3332
PIAS3	0.3334	0.3334	0.3334
JUN	0.4523	0.2142	0.3332
MAPK3	0.4284	0.3809	0.1904
VEGFA	0.1334	0.5334	0.3335
PDGFRA	0.5713	0.1429	0.2857
MAPK9	0.3449	0.3104	0.345
PDGFRB	0.2759	0.3104	0.414
MAPK8	0.1904	0.3809	0.4284
LAMC2	0.276	0.3104	0.414
PIAS2	0.1904	0.4285	0.3808
PIAS1	0.3999	0.4665	0.1332
LAMC1	0.4548	0.3184	0.2274
ITGA2B	0.36	0.32	0.32
ERBB2	0.1428	0.6428	0.2142
NFKBIA	0.3914	0.2174	0.3915
BCL2L1	0.45	0.3	0.25
LAMB4	0.4	0.3	0.3
LAMB3	0.4208	0.2631	0.3156
KRAS	0.2618	0.4047	0.3332
LAMB2	0.44	0.36	0.2
RAC2	0.276	0.3794	0.345
RAC3	0.3335	0.2917	0.3752
RAC1	0.3333	0.5237	0.1428
LAMB1	0.3638	0.3637	0.273
FH	0.28	0.44	0.28

BMP4	0.3122	0.406	0.2809
DVL2	0.3892	0.389	0.2224
DVL3	0.3434	0.281	0.3745
BMP2	0.3057	0.3891	0.3058
TCF7	0.3571	0.2858	0.3571
MAP2K1	0.366	0.3171	0.3172
MAP2K2	0.2665	0.2665	0.4664
TGFBR1	0.35	0.35	0.3
TGFBR2	0.2857	0.4285	0.2856
CREBBP	0.3214	0.4642	0.2142
SMAD4	0.2097	0.3956	0.3959
SMAD3	0.2352	0.3528	0.4116
SMAD2	0.3834	0.3618	0.2556
HGF	0.4093	0.3183	0.273
DVL1	0.5002	0.2779	0.2223
LAMA2	0.4761	0.1904	0.3332
CBLC	0.3822	0.3823	0.2352
LAMA1	0.25	0.3125	0.4375
LAMA4	0.1875	0.375	0.4375
CBLB	0.357	0.3571	0.2856
LAMA3	0.3194	0.2343	0.4472
LAMA5	0.3242	0.3513	0.3241
ETS1	0.3636	0.2727	0.3636
NTRK1	0.2352	0.1764	0.5881
FGF6	0.3571	0.2142	0.4284
FGF5	0.4054	0.2431	0.3512
PPARD	0.2104	0.421	0.3682
FGF8	0.3703	0.3702	0.2591
FGF7	0.4447	0.2778	0.278
FGF9	0.2819	0.333	0.3842
PPARG	0.3683	0.3157	0.3156
MITF	0.3334	0.3334	0.3334

SPI1	0.4285	0.2381	0.3333
MLH1	0.3621	0.3833	0.2556
NFKB1	0.3528	0.3529	0.294
NFKB2	0.3683	0.3158	0.3157
GLI2	0.4001	0.2667	0.3334
GLI3	0.35	0.25	0.4
PTEN	0.28	0.48	0.24
GLI1	0.4999	0.3214	0.1785
FLT3LG	0.2904	0.4195	0.2907
CCNE2	0.3157	0.3683	0.3157
CCNE1	0.3	0.25	0.45
TGFA	0.6524	0.2174	0.1305
HHIP	0.4616	0.1923	0.3463
NOS2A	0.3808	0.2857	0.3332
FGF1	0.225	0.3	0.475
MYC	0.3587	0.3331	0.3074
FGF2	0.4375	0.1875	0.375
FGF3	0.25	0.5625	0.1875
FGF4	0.5002	0.1667	0.3335
AR	0.4058	0.3295	0.2663
CTBP1	0.1904	0.4761	0.3332
CTBP2	0.4829	0.2069	0.3104
TP53	0.4349	0.3044	0.261
RAD51	0.4841	0.3227	0.1937
CCND1	0.2668	0.4001	0.3335
WNT9B	0.2905	0.3872	0.3229
WNT9A	0.3332	0.3665	0.3
BID	0.36	0.32	0.32
APC2	0.3891	0.389	0.2224
PML	0.174	0.5218	0.3045
TFG	0.244	0.4879	0.2684
ZBTB16	0.4642	0.3571	0.1785

KIT	0.2856	0.4285	0.2856
SUFU	0.1819	0.5001	0.3184
SOS1	0.3333	0.3333	0.3333
BCL2	0.2499	0.3214	0.4284
SOS2	0.3228	0.4194	0.2584
WNT8A	0.3679	0.3329	0.2978
WNT8B	0.3214	0.3928	0.2856
FZD9	0.3242	0.3783	0.2971
FZD8	0.4001	0.4001	0.2001
IL6	0.4547	0.2274	0.3183
IL8	0.4443	0.2591	0.296
FZD1	0.3156	0.3683	0.3157
IGF1	0.3044	0.4783	0.2175
BIRC5	0.3479	0.2175	0.4349
FZD3	0.3945	0.342	0.263
FZD2	0.3479	0.2609	0.3915
MAPK10	0.2352	0.3529	0.4116
APPL1	0.36	0.4	0.24
STAT1	0.278	0.3613	0.3614
BIRC3	0.3927	0.2857	0.3213
FZD5	0.38	0.3	0.32
BIRC2	0.3333	0.3999	0.2665
FZD4	0.2424	0.4545	0.303
FZD7	0.3028	0.3491	0.3493
STAT3	0.28	0.32	0.4
FZD6	0.3157	0.263	0.4208
NRAS	0.3587	0.3398	0.3023
WNT7B	0.3528	0.3332	0.3136
RASSF5	0.24	0.36	0.4
GSK3B	0.3794	0.3449	0.276
RASSF1	0.1364	0.4547	0.4095
ARAF	0.44	0.28	0.28

TCEB2	0.45	0.4	0.15
PTCH1	0.3957	0.3026	0.3028
TCEB1	0.3331	0.2998	0.3665
PTCH2	0.3849	0.3079	0.308
WNT7A	0.5	0.35	0.15
FGF19	0.4545	0.3636	0.1818
FGF18	0.3474	0.2388	0.4127
PDGFB	0.2858	0.4859	0.2287
PDGFA	0.3638	0.2728	0.364
FGF14	0.3335	0.1667	0.5004
MMP9	0.4168	0.2084	0.3753
WNT3A	0.2309	0.3077	0.4619
FGF17	0.3153	0.3856	0.2978
STK36	0.4446	0.3334	0.2224
ARNT2	0.3957	0.2794	0.3261
TGFB3	0.2856	0.4047	0.3094
FGF11	0.3331	0.2	0.4666
FOXO1	0.3782	0.2702	0.3512
FGF10	0.3752	0.3751	0.2502
FGF13	0.2857	0.3809	0.3332
FGF12	0.3701	0.3701	0.2591
MMP2	0.6113	0.1667	0.2223
MMP1	0.4	0.35	0.25
TGFB1	0.2381	0.2381	0.5236
TGFB2	0.2	0.35	0.45
WNT2	0.4349	0.1739	0.3915
WNT1	0.4784	0.3479	0.174
WNT4	0.2999	0.3998	0.2998
CDKN2A	0.3	0.65	0.05
WNT3	0.28	0.36	0.36
CDKN2B	0.414	0.4484	0.138
SLC2A1	0.3794	0.3794	0.2415

RALB	0.24	0.52	0.24
CSF3R	0.2856	0.4286	0.2857
RALA	0.4	0.36	0.24
WNT6	0.261	0.4349	0.3045
EGFR	0.3809	0.3333	0.2856
RET	0.2561	0.333	0.4098
RELA	0.2631	0.4209	0.3157
FGF23	0.357	0.1428	0.4998
FADD	0.3335	0.2917	0.3751
FGF22	0.2759	0.2414	0.4829
FGF21	0.3939	0.3333	0.2727
FGF20	0.2728	0.2729	0.4548
STK4	0.2275	0.5001	0.273
CCDC6	0.3183	0.5002	0.1819
EP300	0.348	0.4349	0.2175
HIF1A	0.5182	0.1851	0.296
TRAF1	0.3335	0.2779	0.3891
FGFR2	0.4785	0.4348	0.087
WNT5A	0.3463	0.3846	0.2695
TRAF2	0.3105	0.3794	0.3104
FGFR1	0.45	0.3	0.25
WNT5B	0.3234	0.4117	0.2646
FGFR3	0.3683	0.3684	0.2631
GRB2	0.2105	0.421	0.3683
ITGB1	0.44	0.28	0.28
RBX1	0.357	0.2857	0.357
ARNT	0.3334	0.2667	0.4
PTK2	0.5002	0.2501	0.2502
ITGAV	0.48	0.32	0.2
EGF	0.294	0.2352	0.4705
TRAF6	0.3528	0.5881	0.0588
AXIN2	0.3808	0.3333	0.2856

RUNX1	0.5	0.125	0.375
TRAF5	0.4616	0.1539	0.3849
TRAF4	0.3123	0.2811	0.4059
TRAF3	0.2918	0.5418	0.1668
CSF1R	0.3145	0.3144	0.3715
FN1	0.28	0.2	0.52
AXIN1	0.25	0.35	0.4
EPAS1	0.3386	0.3386	0.3222
MET	0.4139	0.3794	0.207
ITGA2	0.3861	0.4088	0.2044
BRCA2	0.3571	0.3928	0.2499
ITGA3	0.263	0.4736	0.263
CDKN1A	0.4073	0.2963	0.2962
HDAC2	0.2857	0.4286	0.2856
CDKN1B	0.4	0.2	0.4
HDAC1	0.3172	0.2928	0.3904
ITGA6	0.4233	0.3847	0.1925
PLCG1	0.25	0.5	0.25
PLCG2	0.2856	0.3809	0.3332
IKBKG	0.389	0.3334	0.278
IKBKB	0.4	0.32	0.28
ABL1	0.3079	0.4232	0.2695
E2F1	0.4761	0.1905	0.3332
HSP90AB	0.303	0.2727	0.4242
E2F2	0.3914	0.3914	0.2175
E2F3	0.3228	0.355	0.3229
FASLG	0.2961	0.3701	0.3331
AKT1	0.2817	0.3332	0.3842
CDC42	0.2221	0.4074	0.3702
MAX	0.294	0.2941	0.4116
FOS	0.3846	0.2306	0.3842
CASP3	0.52	0.44	0.04

CASP9	0.3717	0.2858	0.343
PAX8	0.4442	0.2962	0.2591
CASP8	0.421	0.1052	0.4734
RHOA	0.4617	0.3462	0.1924
TPR	0.3479	0.348	0.3045
CHUK	0.294	0.3529	0.3528
AKT3	0.3637	0.3183	0.3184
AKT2	0.4573	0.2573	0.2858
PRKCA	0.308	0.4616	0.231
HSP90AA	0.4139	0.2069	0.3795
RALBP1	0.3479	0.4783	0.174
CYCS	0.3872	0.5164	0.0969
RUNX1T1	0.3571	0.3571	0.2856
LEF1	0.2105	0.3683	0.421
CDK6	0.3914	0.3044	0.3045
PRKCG	0.3664	0.3332	0.2997
RB1	0.3663	0.4333	0.1998
CDK4	0.2905	0.4517	0.2583
DAPK2	0.36	0.32	0.32
DAPK3	0.2823	0.3476	0.3692
CDK2	0.357	0.357	0.2856
PRKCB1	0.4285	0.3571	0.2142
DAPK1	0.2963	0.3332	0.3702
SMO	0.3794	0.4139	0.207
CRKL	0.36	0.32	0.32
NCOA4	0.4548	0.3183	0.2274
MDM2	0.4761	0.3809	0.1429
WNT11	0.4761	0.2381	0.2856
GSTP1	0.2631	0.3158	0.4209
DCC	0.4166	0.25	0.3333
CKS1B	0.432	0.3376	0.2295
WNT16	0.348	0.2175	0.435
EGLN3	0.2224	0.389	0.3892
EGLN2	0.3809	0.1904	0.4284
KITLG	0.4669	0.3334	0.2001
EGLN1	0.2501	0.3752	0.3752

CDH1	0.3551	0.2582	0.3872
TCF7L2	0.348	0.3914	0.261
TCF7L1	0.4317	0.2953	0.2725
TPM3	0.4072	0.2592	0.333
IGF1R	0.303	0.4848	0.2121
PIK3R5	0.375	0.3125	0.3125
PIK3R3	0.4	0.35	0.25
FIGF	0.44	0.2	0.36
PIK3R1	0.3172	0.3171	0.366
PIK3R2	0.3874	0.3872	0.2261
APC	0.4549	0.3182	0.2274
CEBPA	0.3332	0.2962	0.37
COL4A4	0.2928	0.244	0.4636
MSH6	0.4183	0.3043	0.279
COL4A2	0.2631	0.421	0.3156
COL4A1	0.3335	0.3334	0.3336
MSH3	0.4442	0.3332	0.222
KLK3	0.4349	0.2609	0.3045
VHL	0.261	0.5219	0.2175
MSH2	0.3637	0.3182	0.3185
FLT3	0.3462	0.0769	0.5771
CBL	0.364	0.2728	0.3639
RAF1	0.3939	0.303	0.303
BAD	0.3463	0.3848	0.2694
RALGDS	0.3463	0.2693	0.3849
COL4A6	0.4441	0.3702	0.185
WNT2B	0.3845	0.3846	0.2307
HSP90B1	0.5237	0.1904	0.2856
FZD10	0.3939	0.2424	0.3636
BAX	0.3094	0.357	0.3332
JAK1	0.3234	0.3528	0.3234
CRK	0.276	0.3794	0.345
